# Association between Admission Hyperglycemia and Culprit Lesion Characteristics in Nondiabetic Patients with Acute Myocardial Infarction: An Intravascular Optical Coherence Tomography Study

**DOI:** 10.1155/2020/1763567

**Published:** 2020-06-20

**Authors:** Jinying Zhou, Zhaoxue Sheng, Chen Liu, Peng Zhou, Jiannan Li, Runzhen Chen, Li Song, Hanjun Zhao, Hongbing Yan

**Affiliations:** ^1^Department of Cardiology, Fuwai Hospital, National Center for Cardiovascular Diseases, Peking Union Medical College and Chinese Academy of Medical Sciences, Beijing, China; ^2^Fuwai Hospital, Chinese Academy of Medical Sciences, Shenzhen, China

## Abstract

**Background:**

Hyperglycemia is frequently observed in acute myocardial infarction (AMI). Diabetes mellitus (DM) patients and non-DM patients have different culprit lesion phenotypes and few data are available on non-DM patients with admission hyperglycemia. Therefore, we aimed to investigate the association between admission hyperglycemia and culprit lesion characteristics using optical coherence tomography (OCT) in AMI patients.

**Methods and Results:**

We consecutively enrolled 434 patients with AMI, and 277 patients were included in analysis: 65.7% (*n* = 182) non-DM patients and 34.3% (*n* = 95) DM patients. We measured acute blood glucose (ABG) and hemoglobin A_1c_ to calculate the acute-to-chronic glycemic ratio (A/C). Then, we grouped non-DM patients into tertiles of A/C. OCT-based culprit lesion characteristics were compared across A/C tertiles in non-DM patients and between DM and non-DM patients. Non-DM patients had fewer lipid-rich plaques (52.7% versus 68.4%, *p* = 0.012) and thin-cap fibroatheroma (TCFA) (19.8% versus 34.7%, *p* = 0.006) than DM patients but similar prevalence of plaque rupture (47.3% versus 56.8%, *p* = 0.130). Non-DM patients with the highest A/C tertile had the highest prevalence of plaque rupture (*p*_for trend_ = 0.002), lipid-rich plaque (*p*_for trend_ = 0.001), and TCFA (*p*_for trend_ = 0.003). A/C > 1.22 but not ABG > 140 mg/dl predicted a high prevalence of plaque rupture, lipid-rich plaque, and TCFA in non-DM patients.

**Conclusions:**

In AMI patients without DM, admission hyperglycemia is associated with vulnerable culprit lesion characteristics, and A/C is a better predictor for vulnerable culprit plaque characteristics than ABG. These results call for a tailored evaluation and management of glucose metabolism in nondiabetic AMI patients. This trial is registered with NCT03593928.

## 1. Introduction

Diabetes mellitus (DM) in general increases vascular complications, including coronary heart disease, ischemic stroke, and vascular deaths [1]. DM patients have a high prevalence of multivessel disease and an accelerated atherosclerotic progression related to the glucose level [2]. Optical coherence tomography (OCT) allows accurate evaluation of coronary atherosclerotic plaques *in vivo*. Although a pilot study including 63 participants with coronary artery diseases (CAD) found no significant difference in plaque characteristics between the DM group and the non-DM group [3], recent studies reported conflicting results that diabetic individuals with CAD may have more calcification [4, 5] or lipid-rich plaques [6–8]. Additionally, individuals with hemoglobin A_1c_ (HbA_1c_) ≥8% had the highest prevalence of thin-cap fibroatheroma (TCFA) [6]. These results indicate that the glucose level has an impact on coronary plaque characteristics, but it remains unclear whether such impact exists in non-DM individuals with CAD.

Acute hyperglycemia at admission is frequently observed in acute myocardial infarction (AMI) patients without DM [9–11]. However, little is known about the relationship between admission hyperglycemia and culprit plaque characteristics. Besides, some of these patients have undiagnosed DM [12, 13] which probably affects acute blood glucose (ABG) at admission. Thus, the acute-to-chronic glycemic ratio (A/C) [14], which is calculated by dividing ABG by chronic blood glucose (CBG) estimated from HbA_1c_ [15], has been used to reflect relative hyperglycemia at admission.

In this study, we aim to investigate whether admission hyperglycemia in nondiabetic patients with AMI is associated with vulnerable culprit lesion characteristics such as plaque rupture, lipid-rich plaque, and TCFA. In addition, we compared different culprit plaque characteristics between diabetic and nondiabetic patients and evaluated two definitions for admission hyperglycemia for predicting vulnerable culprit plaque characteristics in AMI patients.

## 2. Materials and Methods

### 2.1. Study Population

The Optical Coherence Tomography Examination in Acute Myocardial Infarction (OCTAMI, NCT03593928) is a prospective, single-center, observational registry. In brief, consecutive patients at Fuwai Hospital were screened for OCT examination. The major inclusion criteria were (1) age ≥ 18 years, (2) presented with ST-segment elevated myocardial infarction (STEMI), and (3) referred to primary percutaneous coronary intervention. The major exclusion criteria were (1) cardiogenic shock; (2) history of coronary artery bypass graft; (3) left main diseases, extremely tortuous or heavily calcified vessels; and (4) inability to obtain Thrombolysis in Myocardial Infarction Flow grade ≥ 2. STEMI was defined as clinical symptoms, elevated troponin I level, and typical ST-segment elevation on electrocardiogram. In addition to the exclusion criteria of the OCTAMI registry, patients were excluded from the current study if (1) the culprit lesions were in-stent restenosis, coronary spasm, coronary embolism, or calcified nodule and (2) HbA_1c_ results were missing. All treatments were as per standard of care. Culprit vessel was determined primarily by coronary angiography and corroborated with electrocardiogram and echocardiographic results or ventriculographic assessments. All enrolled patients provided written informed consent. The study complied with the principles of the Declaration of Helsinki and was approved (no. 2017-866) by the Review Board of Fuwai Hospital.

### 2.2. Blood Glucose Measurements and Diagnosis of Diabetes

ABG was measured upon admission. HbA_1c_ was measured during hospital stay. DM was diagnosed if a patient had a history of diabetes, received insulin or oral hypoglycemic agents, or had HbA_1c_ ≥ 6.5% (48 mmol/mol). CBG was estimated from HbA_1c_ according to the following formula [15]: Estimated CBG [mmol/l] = {28.7 × HbA1c [%or (mmol/mol)] − 46.7[mg/dl]}/18. A/C was calculated as the ratio of ABG to estimated CBG. Two definitions for admission hyperglycemia were compared: (1) ABG ≥ 140 mg/dl (7.8 mmol/l) [16] and (2) A/C higher than the upper tertile cut-off of the current study population.

### 2.3. OCT Image Acquisition and Analysis

OCT examinations were performed as previously described [17, 18]. Briefly, OCT images of the culprit lesions were acquired using the frequency-domain OCT system (ILUMIEN OPTIS™, St. Jude Medical/Abbott, St. Paul, MN, USA) and a dragonfly catheter (Lightlab Imaging, Inc., Westford, MA, USA). Thrombus aspiration and/or gentle predilation were applied as per need. The total length of OCT pullback was 75 mm. All OCT images were submitted to an offline St. Jude OCT Offline Review Workstation and analyzed by three independent investigators blinded to angiographic and clinical data. Any discordance was resolved by consensus. The culprit plaque was defined as the segment centered on the culprit lesion and extending bilaterally to ≥5 mm of normal vessel segment [19].

Definitions of image characteristics on OCT were based mainly on established consensus [20], and details of these definitions have been described previously [18]. Lipid was defined as a low-signal region with a poorly defined or diffuse border. Lipid arc was measured at 1 mm intervals across the entire lesion, and the largest arc was recorded. A plaque with a maximal lipid arc > 90° was defined as a lipid-rich plaque (Figure S1b); otherwise, it was defined as a fibrous plaque (Figure S1a). Fibrous cap thickness (FCT) was measured in triplicate at the thinnest part of the fibrous cap of the culprit plaque, and the average value was calculated and reported. TCFA was defined as a lipid-rich plaque with FCT < 65 *μ*m. Plaque rupture was defined as disruption of the fibrous cap with clear cavity formation (Figure S1c). Plaque erosion was defined based on evidence of thrombus on an irregular luminal surface without evidence of cap rupture in multiple adjacent frames (Figure S1d). Calcification was defined as signal-poor or heterogeneous regions with well-delineated borders (Figure S1e). Microvessels were defined as signal-poor, tubular structures without a connection to the vessel lumen in more than three consecutive cross-sectional images (Figure S1f). Cholesterol crystals were defined as linear structures with high backscatter within the plaque (Figure S1g). Macrophage infiltration was defined as signal rich, distinct, or confluent punctate regions above the intensity of background speckle noise with backward shadowing, usually located at the boundary between the fibrous cap and inner lipid core (Figure S1h). Thrombus was defined as an irregular mass floating in the lumen or adjacent to the luminal surface. The minimal lumen area (MLA) was the smallest lumen area within the length of the target lesion.

### 2.4. Statistical Analyses

Continuous variables with normal distribution are presented as mean ± SD and compared between groups using the independent *t*-test; nonnormal variables were reported as median (interquartile range) and compared between groups using the Kruskal-Wallis test. Categorical variables are presented as number (%) and compared between two groups by the *χ*^2^ test or the Fisher exact test. The *p*_for trend_ were determined with a Wilcoxon type test for continuous variables and linear-by-linear association for categorical variables across ordered three A/C tertile groups in non-DM patients. Bivariate correlations between OCT measurements including FCT, MLA, and maximal lipid arc (as continuous variables) and metabolic variables (as continuous variables) were analyzed using Spearman's correlation. Univariable and multivariable logistic regression analyses were applied to determine associations of OCT categorical characteristics (plaque rupture, lipid-rich plaque, and TCFA) and admission hyperglycemia. For admission hyperglycemia, we tested two different definitions by either ABG or A/C, respectively, in univariable and multivariable logistic regression analyses. Covariates adjusted in the multivariable logistic regression model were age, sex, body mass index, current smoking, hypertension, baseline cardiac troponin I (cTnI), baseline creatine kinase MB (CK-MB), total cholesterol (TC), triglyceride (TG), low-density lipoprotein cholesterol (LDL-C) l, high-density lipoprotein cholesterol (HDL-C), high-sensitive C reactive protein (hsCRP), estimated glomerular filtration rate (eGFR), previous statin usage, and time from symptom onset. A two-tailed *p* value < 0.05 was considered statistically significant. All analyses were performed using SPSS Statistics 24.0 software (IBM Corp, Armonk, NY) except that the Wilcoxon type test was performed using the R package rawr (version 0.9.1, Robert Redd, https://github.com/raredd/rawr; R Foundation for Statistical Computing, Vienna, Austria).

## 3. Results

**Figure 1:**
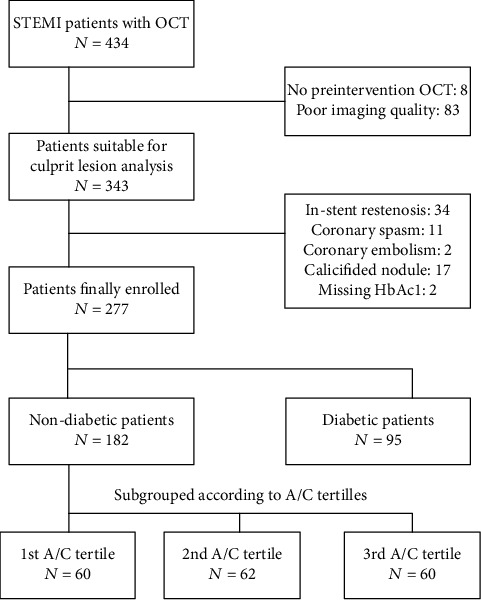
Flow chart. A/C: acute-to-chronic glycaemia ratio; HbA1c: hemoglobin A1c; OCT: optical coherence tomography; STEMI: ST-segment elevated myocardial infarction.

Patients' baseline characteristics are summarized in Table 1. In total, 434 STEMI patients undergoing OCT examination from March 2017 to March 2019 were enrolled. Of these, 157 were excluded because of lack of preintervention OCT (*n* = 8), poor imaging quality (*n* = 83), in-stent restenosis (*n* = 34), coronary spasm (*n* = 11), coronary embolism (*n* = 2), calcified nodule (*n* = 17), and missing HbA_1c_ data (*n* = 2) (Figure 1). Table S1 (see supplementary material) shows the comparisons of baseline characteristics between included and excluded patients. Finally, 277 patients were included in the analysis, with 182 (65.7%) nondiabetic patients and 95 (34.3%) diabetic patients. Non-DM patients had lower ABG (132.6 mg/dl (interquartile range 119.5–146.3) versus 211.5 mg/dl (177.2–271.3), *p* < 0.001) and lower HbA_1c_ (5.7% (5.5–5.9) versus 7.9% (7.0–9.3), *p* < 0.001) than DM patients but similar A/C (1.15 (1.03–1.27) versus 1.21 (1.03–1.38), *p* > 0.05). DM patients were heavier (*p* < 0.05) and had more multivessel disease (*p* < 0.05) than non-DM patients. Non-DM patients were further grouped by tertiles of A/C, with a lower tertile cut-off of 1.08 and upper tertile cut-off of 1.22. In non-DM patients, those in the highest A/C tertile group were admitted to the hospital in the shortest time since symptom onset (*p*_for trend_ = 0.005). Across three A/C tertiles in non-DM patients, there was a decreasing trend of cTnI (*p*_for trend_ = 0.009) and CK-MB (*p*_for trend_ = 0.038). However, after adjustment for time from symptom onset, there was no linear association between A/C tertiles and cTnI or between A/C tertiles and CK-MB (Table S2).

**Figure 2:**
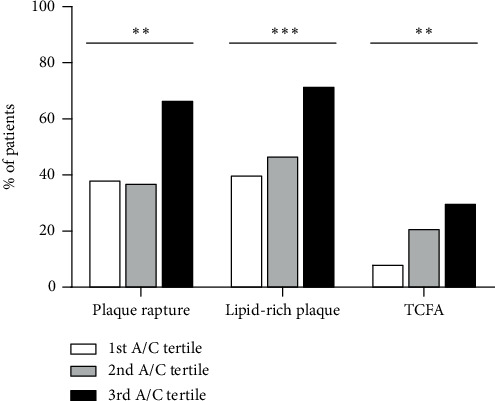
Comparison of vulnerable culprit lesion characteristics among A/C tertiles in nondiabetic patients with AMI, *n* = 182. White bars: first A/C tertile, *n* = 60; grey bars: second A/C tertile, *n* = 62; black bars: third A/C tertile, *n* = 60. ^∗∗^*p*_for trend_ ≤ 0.01, ^∗∗∗^*p*_for trend_ ≤ 0.001.

Table 2 shows culprit lesion characteristics on OCT examination. Non-DM patients in the third A/C tertile group had the highest prevalence of plaque rupture (66.7% versus 37.1% versus 38.3% from the third to the first tertile, *p*_for trend_ = 0.002), lipid-rich plaque (71.7% versus 46.8% versus 40.0% from the third to the first tertile, *p*_for trend_ = 0.001), and TCFA (30.0% versus 21.0% versus 8.3% from the third to the first tertile, *p*_for trend_ = 0.003) (Figure 2). Consistent with this, patients in the third A/C tertiles had the smallest FCT (median 90.0 mm versus 110.0 mm versus 110.0 mm from the third to the first tertile, *p*_for trend_ = 0.010). Rates of macrophage infiltration, microvessels, cholesterol crystal, calcification, and thrombus were similar among the A/C tertiles in nondiabetic patients. While comparing the third A/C tertile group of non-DM patients with DM patients, the prevalence of plaque rupture (66.7% versus 56.8%, *p*_3rd versus DM_ = 0.222), lipid-rich plaque (71.7% versus 68.4%, *p*_3rd versus DM_ = 0.669), and TCFA (30.0% versus 34.7%, *p*_3rd versus DM_ = 0.406) was similar. The prevalence of calcification in DM patients was significantly higher than that of the third A/C tertile group of non-DM patients (*p* = 0.002) but only numerically higher than that of the other two A/C tertile groups of non-DM patients. On the other hand, the lowest A/C tertile of non-DM patients, compared with DM patients, had fewer macrophage infiltration (*p* = 0.049) and smaller FCT (*p* = 0.013). Overall, the DM group, compared with the non-DM group, had a significantly higher prevalence of lipid-rich plaque (68.4% versus 52.7%, *p* = 0.012), TCFA (34.7% versus 19.8%, *p* = 0.006), cholesterol crystal (12.6% versus 5.5%, *p* = 0.037), and calcification (62.1% versus 46.2%, *p* = 0.012). However, the prevalence of plaque rupture was similar between DM patients and non-DM patients.

Table 3 shows the correlations between quantitative OCT measurements and glycemic markers and other laboratory variables in non-DM patients and DM patients. In non-DM patients, ABG and A/C were correlated with FCT (*r* = −0.149, *p* = 0.045; *r* = −0.206, *p* = 0.005, respectively). On the other hand, low-density lipoprotein cholesterol (LDL-C) and the estimated glomerular filtration rate (eGFR) were correlated with MLA (*r* = −0.164, *p* = 0.027; *r* = −0.229, *p* = 0.002, respectively) and lipid arc (*r* = 0.228, *p* = 0.037; *r* = 0.226, *p* = 0.039, respectively). In DM patients, no glycemic index was correlated with FCT, but total cholesterol (TC) and LDL-C were correlated with FCT (*r* = −0.216, *p* = 0.036; *r* = −0.252, *p* = 0.014, respectively). All those correlations were significant but weak.

**Figure 3:**
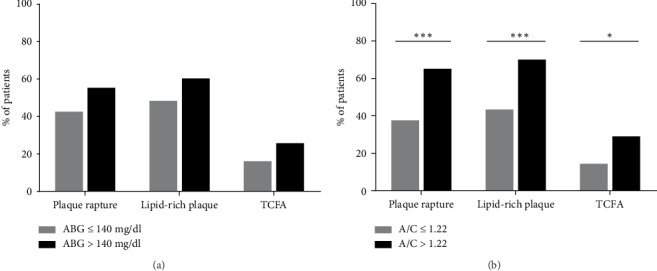
Comparisons of vulnerable culprit lesion characteristics by different hyperglycemia criteria in non-diabetic patients with AMI, *n* = 182. (a) Hyperglycemia defined as A/C > 1.22; (b) hyperglycemia defined as ABG > 140 mg/dl. Gray bars: no hyperglycemia; black bars: hyperglycemia. Significant difference: ^∗^*p* ≤ 0.05, ^∗∗∗^*p* ≤ 0.001.

Table 4 shows results from univariate and multivariate logistic regression analyses of admission hyperglycemia for predicting plaque rupture, lipid-rich plaque, and TCFA in STEMI patients with or without DM, respectively. In non-DM patients, after adjustment for covariates, A/C > 1.22 remained predictive for plaque rupture (hazard ratio (HR) 3.11, 95% confidence interval (CI) 1.48–6.55, *p* = 0.003), lipid-rich plaque (HR 2.94, 95% CI 1.36–6.35, *p* = 0.006), and TCFA (HR 2.40, 95% CI 1.01–5.72, *p* = 0.049) but ABG > 140 mg/dl was not predictive for plaque characteristics (Figure 3). In DM patients, both A/C > 1.22 and ABG > 140 mg/dl had no predictive value for plaque characteristics.

## 4. Discussion

Our study demonstrated that (1) DM patients with STEMI had a significantly higher prevalence of lipid-rich plaque, TCFA, cholesterol crystal, and calcification than non-DM patients; (2) non-DM STEMI patients with admission hyperglycemia had a higher prevalence of plaque rupture, lipid-rich plaque, and TCFA at the culprit lesion than those without admission hyperglycemia; (3) admission hyperglycemia in non-DM patients defined as A/C > 1.22 had a better predictive value for plaque rupture, lipid-rich plaque, and TCFA than that defined as ABG > 140 mg/dl.

### 4.1. Culprit Lesion Characteristics in Diabetic STEMI

Different coronary and plaque characteristics between DM and non-DM patients have been reported. Previous studies showed larger lipid burden [21, 22], more plaque ruptures, and more TCFAs in diabetic patients than in nondiabetic patients [23]. Nicholls et al. [2] reported a strong relationship between percent atheroma volume and HbA_1c_. Several OCT studies [3–6, 8] had a limited sample size. The largest study [7] enrolled 322 patients with acute coronary syndrome and reported more lipid-rich plaque in the culprit lesion in the DM group than in the non-DM group. In addition, no difference in plaque rupture was observed between the groups [7]. Consistent with their study, our study showed more lipid-rich plaque and TCFA in DM patients than non-DM STEMI patients but a similar prevalence of plaque rupture in culprit lesion.

### 4.2. Culprit Lesion Characteristics in Nondiabetic STEMI

To our knowledge, this is the first study to compare plaque characteristics in non-DM patients according to A/C tertiles. The current study showed that non-DM patients with the highest A/C tertile had a higher prevalence of plaque rupture, lipid-rich plaque, and TCFA than those in the other A/C tertiles. In consistence, non-DM patients with the highest A/C tertile also had the smallest FCT. Surprisingly, the prevalence of plaque rupture, lipid-rich plaque, and TCFA was similar between non-DM patients in the highest A/C tertile group and the DM group. Another recent study [18] from our group reported that AMI patients with increased duration of DM had a higher prevalence of plaque rupture, lipid-rich plaque, and TCFA along with higher HbA1c levels than those with short DM duration. In combination, these results suggested that hyperglycemia has a strong association with vulnerable plaque characteristics, namely, plaque rupture, lipid-rich plaque, and TCFA, regardless of DM status. It is worth mentioning that pretreatment with statin before acute coronary syndrome reduces not only the presentation of STEMI but also the prevalence of ruptured plaque and TCFA [24]. In the present study population, 18.7% of non-DM patients and 13.7% DM patients were on statin therapy before the index STEMI (*p* = 0.293). Moreover, there was no difference of statin therapy across A/C tertiles in non-DM patients. Thus, we do not consider statin pretreatment as a confounder between A/C tertiles and plaque rupture or TCFA in non-DM patients.

Calcification is a long-term pathological change, and hyperglycemia promotes vascular calcification via multiple mechanisms such as oxidative stress, endothelial dysfunction, and advanced accumulation of glycation end products [25]. Moreover, race, sex, and age affect the prevalence of vascular calcification [26]. A previous OCT study showed an impact of chronic kidney diseases on coronary calcification [27]. The present study showed similar prevalence of calcification across three A/C tertiles but significantly fewer calcification in non-DM patients with the highest A/C than in DM patients, which may result from the adjustment of those aforesaid confounders.

Coronary macrophage infiltration is another risk characteristic reflecting inflammatory level [22]. MacNeill et al. [28] reported more macrophage infiltration in unstable patients and culprit lesion than in stable patients and nonculprit lesions, respectively. The present study showed no difference in macrophage infiltration among A/C tertiles in non-DM patients or between the DM and non-DM groups. However, those non-DM patients with the lowest A/C tertile had fewer macrophage infiltration than DM patients.

Although we could not make a cause-effective conclusion between admission hyperglycemia and culprit lesion characteristics, our hypothesis is that admission hyperglycemia represents severe myocardial infarction attack. Plaque rapture causes the sudden onset of acute myocardial infarction [29], and hormonal response to stress has a significant impact on hyperglycemia [30]. Then, hyperglycemia accelerate the vicious cycle of myocardial infarction by attenuated endothelium vasodilation [31], activated platelets [32], enhanced leukocyte accumulation [33], elevated inflammatory level [34], and increased thrombin generation potential [35]. In addition, previous studies reported that glycemic variability can promote atherosclerosis [36–39]. We also inferred that recurrent transient hyperglycemia before an index event has an accumulating impact on coronary atherosclerosis as well, but future studies are needed for evidence.

### 4.3. A/C as a Predictor for Plaque Vulnerability in Nondiabetic AMI

Numerous glycemic metrics are available in clinical practice, but only a few are practical in a real-world acute setting of myocardial infarction. An early study by Capes et al. [40] reported an increased risk of death after AMI in all patients with high-glucose concentration on admission, which was described as stress hyperglycemia. However, it is plausible that AMI patients with the same ABG might have a different chronic glycemic metabolic status. Although chronic glucose level cannot be directly detected in AMI patients, it can be estimated from HbA_1c_ using the formula proposed by Nathan et al. [15]. Relative hyperglycemia or the stress hyperglycemia ratio, defined as ABG divided by estimated CBG (acute-to-chronic glucose ratio, A/C), was first proposed by Roberts *et al.* [14] as an improved biomarker of critical illness. Recent studies reported a prominent predictive value of A/C for prognosis in AMI patients [41, 42]. On the other hand, high-risk plaque is another independent prognostic predictor in patients with CAD (coronary artery disease) [43–46]. Thus, in AMI patients without DM, there may be an interrelation among acute hyperglycemia, vulnerable plaque, and poor prognosis. Our study showed a weak but significant correlation between ABG or A/C with FCT in nondiabetic patients.

Recent studies are exploring patient-tailored treatment strategy based on culprit lesion characteristics. EROSION Study (Effective Anti-Thrombotic Therapy Without Stenting: Intravascular Optical Coherence Tomography-Based Management in Plaque Erosion) [47] was the first study to suggest that patient with acute coronary syndrome due to plaque erosion can be managed by effective antiplatelet therapy without stent implantation. Torii et al. reported the impact of plaque type on stent strut [48]. However, high price and technical safety concerns restrict the application of OCT to a selected population of AMI, surrogates for predicting culprit lesions are needed in clinical practice. Our results showed that A/C ratio had a significant association with vulnerable plaque characteristics. Moreover, admission hyperglycemia defined by A/C > 1.22 was associated with a high risk of plaque rupture, lipid-rich plaque, and TCFA, but admission hyperglycemia defined by ABG > 140 mg/dl had no predictive value. Thus, A/C had a better predictive ability than ABG alone for plaque vulnerability, which might be an important message to physicians in clinical practice.

### 4.4. Study Limitations

Some limitations warrant mention. First, this is an observational study with prospectively enrolled patients and retrospectively collected data, and therefore, no cause-effect relationship between hyperglycemia and vulnerable plaque characteristics could be established. Second, OCT examination is restricted to patients with relatively stable hemodynamics for ethical and safety reasons, and thus, selection bias could not be eliminated which undoubtedly contributed to the percent (50.5%) of plaque rupture in our study population being much lower than that (70%) reported in a meta-analysis by Iannaccone et al. [49]. Third, undiagnosed DM is not rare in AMI. Thus, we carefully screened for DM history and HbA_1c_. In addition, we applied A/C in the non-DM group, which had taken into consideration both ABG and CBG and were available in an acute setting of AMI. Fourth, due to the limited number of patients in our study cohort, we cannot extend our conclusions to all AMI patients. Therefore, further studies are needed.

## 5. Conclusions

This study demonstrated that non-DM patients with admission hyperglycemia had a higher prevalence of vulnerable culprit plaque characteristics than those without admission hyperglycemia. Moreover, A/C was more valuable than ABG in predicting culprit plaque vulnerability in non-DM patients with AMI. These findings highlight the important role of admission hyperglycemia in non-DM patients with AMI. Future studies are needed to improve the glucose evaluation and management strategies which may consequently improve clinical outcomes in these patients.

## Figures and Tables

**Table 1:** Baseline characteristics of the study population.

	Non-DM by A/C tertiles					DM	
		<1.08 *N* = 60	1.08-1.22 *N* = 62	>1.22 *N* = 60	*p*_for trend_	*N* = 95	*p*_non−DM versus DM_
Age	57.6 ± 11.6	58.3 ± 12.3	57.3 ± 12.0	57.1 ± 10.6	0.438	57.1 ± 11.5	0.725
BMI (kg/m^2^)	25.8 ± 3.9	26.4 ± 5.3	25.5 ± 3.2	25.6 ± 2.7	0.467	26.8 ± 3.3	0.038
Men	150 (82.4)	53 (88.3)	49 (79.0)	48 (80.0)	0.232	77 (81.1)	0.779
Smoking	125 (68.7)	46 (76.7)	42 (67.7)	37 (61.7)	0.077	67 (70.5)	0.752
Time from symptom onset (hour)	6.1 (2.9, 13.8)	7.3 (3.8, 14.7)	7.9 (3.3, 16.9)	3.7 (2.3, 8.0)	0.005	5.3 (2.7, 11.5)	0.576
Hypertension	101 (55.5)	36 (60.0)	31 (50.0)	34 (56.7)	0.714	61 (64.2)	0.162
Dyslipidemia	166 (91.2)	54 (90.0)	55 (88.7)	57 (95.0)	0.335	85 (89.5)	0.638
Prior PCI	12 (6.6)	3 (5.0)	5 (8.1)	4 (6.7)	0.714	9 (9.5)	0.390
ABG (mg/dl)	132.6 (119.5, 146.3)	116.4 (108.5, 124.3)	133.5 (126.1, 142.0)	154.6 (139.0, 177.2)	<0.001	211.5 (177.2, 271.3)	<0.001
HbA1c (%)	5.7 (5.5, 5.9)	5.8 (5.6, 6.0)	5.7 (5.5, 5.9)	5.6 (5.4, 5.8)	<0.001	7.9 (7.0, 9.3)	<0.001
A/C	1.15 (1.03, 1.27)	0.97 (0.92, 1.03)	1.15 (1.10, 1.18)	1.34 (1.27, 1.46)	<0.001	1.21 (1.03, 1.38)	0.127
cTnI	3.9 ± 8.6	4.9 ± 10.4	4.1 ± 8.8	2.5 ± 6.2	0.009	3.4 ± 7.1	0.583
CK-MB	70.9 ± 104.1	79.1 ± 109.7	82.4 ± 107.2	51.0 ± 93.4	0.038	56.1 ± 86.2	0.200
WBC (10^6^/l)	10.6 ± 3.2	10.2 ± 3.0	10.3 ± 3.4	11.3 ± 3.2	0.082	10.6 ± 3.0	0.995
Hb (g/l)	147.1 ± 16.7	147.3 ± 17.4	147.3 ± 16.8	146.5 ± 16.2	0.993	149.1 ± 15.9	0.331
TC (mg/dl)	168.2 ± 40.0	168.2 ± 40.8	172.5 ± 37.1	163.8 ± 42.1	0.858	176.4 ± 40.2	0.108
TG (mg/dl)	135.9 ± 93.0	134.5 ± 79.2	130.5 ± 80.3	142.7 ± 116.3	0.988	185.9 ± 127.6	<0.001
HDL-C (mg/dl)	43.1 ± 14.5	42.6 ± 21.2	43.5 ± 9.8	43.1 ± 9.9	0.074	40.9 ± 8.8	0.189
LDL-C (mg/dl)	107.1 ± 33.1	106.9 ± 35.1	110.5 ± 31.7	103.8 ± 32.7	0.888	111.2 ± 33.3	0.327
Lp(a) (mg/l)	179.8(84.0, 394.0)	144.1(66.5, 318.1)	207.3(99.0, 458.0)	194.0(102.8, 357.0)	0.130	130.2 (54.5, 309.8)	0.070
eGFR (mL/min/1.73 m^2^)	94.6 ± 33.3	102.1 ± 40.9	91.5 ± 29.3	90.2 ± 27.6	0.183	99.5 ± 27.1	0.211
Hs-CRP (mg/l)	5.3 (2.5, 10.9)	5.6 (2.9, 11.1)	5.9 (2.8, 10.4)	4.6 (1.8, 11.0)	0.337	6.4 (2.7, 10.3)	0.748
LVEF (%)	54.7 ± 6.5	55.3 ± 5.6	55.6 ± 6.9	53.2 ± 6.9	0.092	55.7 ± 5.4	0.208
Aspirin	63 (34.6)	25 (41.7)	19 (30.6)	19 (31.7)	0.251	41 (43.2)	0.163
P2Y12 inhibitor	42 (23.1)	17 (28.3)	15 (24.2)	10 (16.7)	0.130	28 (29.5)	0.245
Statin	34 (18.7)	11 (18.3)	12 (19.4)	11 (18.3)	1.000	13 (13.7)	0.293
Culprit vessels					0.240		0.245
LAD	88 (48.4)	28 (46.7)	26 (41.9)	34 (56.7)		47 (49.5)	
LCX	23 (12.6)	5 (8.3)	13 (21.0)	5 (8.3)		6 (6.3)	
RCA	71 (39.0)	27 (45.0)	23 (37.1)	21 (35.0)		42 (44.2)	
LM disease	5 (2.7)	3 (5.0)	1 (1.6)	1 (1.7)	0.265	1 (1.1)	0.358
MVD	127 (69.8)	43 (71.7)	41 (66.1)	43 (71.7)	1.000	77 (81.1)	0.043
Pre-TIMI flow					0.763		0.480
0	124 (68.1)	39 (65.0)	43 (69.4)	42 (70.0)		56 (58.9)	
1	9 (4.9)	4 (6.7)	3 (4.8)	2 (3.3)		5 (5.3)	
2	15 (8.2)	6 (10.0)	4 (4.8)	5 (8.3)		10 (10.5)	
3	34 (18.7)	11 (18.3)	12 (19.4)	11 (18.3)		24 (25.3)	
Aspiration	107 (58.8)	35 (58.3)	37 (59.7)	35 (58.3)	1.000	65 (68.4)	0.117
Predilation	148 (81.3)	48 (80.0)	50 (80.6)	50 (83.3)	0.640	72 (75.8)	0.280

Data shown are number (%), median (25th, 75th percentiles), or mean ± SD. ABG: acute blood glucose; A/C: acute-to-chronic glycemic ratio; BMI: body mass index; CK-MB: creatine kinase MB; cTnI: cardiac troponin I; DM: diabetes mellitus; eGFR: estimated glomerular filtration rate; HbA1c: glycosylated hemoglobin; HDL-C: high-density lipoprotein cholesterol; Hs-CRP: high-sensitivity C-reactive protein; LAD: left anterior descending artery; LCX: left circumflex artery; LM: left main coronary artery; LVEF: left ventricular ejection fraction; MVD: multivessel disease; non-DM: patients without diabetes mellitus; PCI: percutaneous coronary intervention; RCA: right coronary artery; TC: total cholesterol; TG: triglyceride; TIMI: thrombolysis in myocardial infarction; WBC; white blood cell.

**Table 2:** Culprit lesion characteristics of the study population by optical coherence tomography.

	Non-DM patients by A/C tertiles				*p*_for trend_	DM	*p*_non−DM_ versus DM	*p*_1st_ versus DM	*p*_2nd_ versus DM	*p*_3rd_ versus DM
	*N* = 182	<1.08 *N* = 60	1.08-1.22 *N* = 62	>1.22 *N* = 60		*N* = 95				
Plaque morphology					0.002		0.130	0.025	0.016	0.222
Plaque rupture	86 (47.3)	23 (38.3)	23 (37.1)	40 (66.7)		54 (56.8)				
Plaque erosion	96 (52.7)	37 (61.7)	39 (62.9)	20 (33.3)		41 (43.2)				
Plaque type					0.001		0.012	<0.001	0.007	0.669
Lipid-rich plaque	96 (52.7)	24 (40.0)	29 (46.8)	43 (71.7)		65 (68.4)				
Fibrous plaque	86 (47.3)	36 (60.0)	33 (53.2)	17 (28.3)		30 (31.6)				
TCFA	36 (19.8)	5 (8.3)	13 (21.0)	18 (30.0)	0.003	33 (34.7)	0.006	<0.001	0.022	0.406
Macrophage infiltration	94 (51.6)	26 (43.3)	34 (54.8)	34 (56.7)	0.145	57 (60.0)	0.185	0.049	0.620	0.739
Microvessels	32 (17.6)	13 (21.7)	7 (11.3)	12 (20.0)	0.811	17 (17.9)	0.948	0.677	0.365	0.833
Cholesterol crystal	10 (5.5)	3 (5.0)	4 (6.5)	3 (5.0)	1.000	12 (12.6)	0.037	0.118	0.211	0.118
Calcification	84 (46.2)	29 (48.3)	33 (53.2)	22 (36.7)	0.201	59 (62.1)	0.012	0.092	0.270	0.002
Thrombus	176 (96.7)	58 (96.7)	59 (95.2)	59 (98.3)	0.610	94 (98.9)	0.259	0.560	0.301	0.599
FCT (*μ*m)	100.0 (70.0, 130.0)	110.0 (80.0, 140.0)	110.0 (70.0, 140.0)	90.0 (60.0, 115.0)	0.010	90.0 (60.0, 120.0)	0.067	0.013	0.052	0.840
Maximal lipid arc (°)	360 (242, 360)	322.3 (241.5, 360.0)	339.5 (235.0, 360.0)	360.0 (262.0, 360.0)	0.173	360.0 (242.0, 360.0)	0.295	0.184	0.169	0.752
MLA (mm^2^)	1.73 (1.36, 2.23)	1.55 (1.28, 2.39)	1.74 (1.44, 2.17)	1.75 (1.35, 2.32)	0.654	1.72 (1.50, 2.14)	0.381	0.388	0.414	0.744

Data shown are number (%) or median (25th percentile, 75th percentile). DM: diabetes mellitus; FCT: fibrous cap thickness; MLA: minimal lumen area; non-DM: patients without diabetes mellitus; TCFA: thin-cap fibroatheroma.

**Table 3:** Association between optical coherence tomography measurements and metabolic variables of the study population.

	ABG	HbA1c	A/C	TC	TG	HDL-C	LDL-C	Lp (a)	Hs-CRP	eGFR
Non-DM group										
FCT, *n* = 182	-0.149^∗^	0.091	-0.206^†^	-0.138	-0.045	-0.019	-0.122	0.097	0.143	0.065
Lipid arc, *n* = 84^‡^	-0.038	0.006	-0.019	0.193	-0.026	0.092	0.228^∗^	0.136	-0.137	0.226^∗^
MLA, *n* = 182	0.050	0.007	0.034	-0.082	0.028	0.112	-0.164^∗^	0.035	0.025	-0.229^†^
DM group										
FCT, *n* = 95	0.157	0.158	-0.029	-0.216^∗^	0.012	0.031	-0.252^∗^	0.194	0.154	0.108
Lipid arc, *n* = 38^‡^	-0.022	0.011	0.071	0.073	-0.116	0.059	0.079	-0.096	0.014	-0.105
MLA, *n* = 95	0.040	0.072	0.005	-0.018	0.045	-0.039	-0.049	-0.130	0.062	0.072

^∗^ *p* < 0.05; ^†^*p* < 0.01. ^‡^Individuals with lipid arc = 360° were excluded, as truncated data is refused by Spearman's correlations. DM: diabetes mellitus.

**Table 4:** Logistic regression analysis for plaque rupture, lipid plaque, and thin-cap fibroatheroma.

		Univariable	*p* values	Multivariable	*p* values
		OR (95% CI)		OR (95% CI)	
Non-DM	Plaque rupture				
*N* = 182	ABG > 140 m/dl	1.67 (0.90-3.11)	0.105	1.65 (0.80-3.38)	0.174
	A/C > 1.22	3.11 (1.63-5.91)	0.001^†^	3.11 (1.48-6.55)	0.003^†^
	Lipid-rich plaque				
	ABG > 140 m/dl	1.62 (0.87-3.03)	0.130	1.69 (0.80-3.56)	0.169
	A/C > 1.22	3.07 (1.59-5.91)	0.001^†^	2.94 (1.36-6.35)	0.006^†^
	Thin-cap fibroatheroma				
	ABG > 140 m/dl	1.80 (0.85-3.78)	0.124	1.86 (0.80-4.35)	0.151
	A/C > 1.22	2.40 (1.14-5.04)	0.021^∗^	2.40 (1.01-5.72)	0.049^∗^
DM	Plaque rupture				
N =95	ABG>140 m/dl	2.14 (0.56-8.16)	0.264	3.50 (0.59-20.70)	0.167
	A/C >1.22	1.28 (0.57-2.89)	0.556	1.00 (0.37-2.72)	0.994
	Lipid-rich plaque				
	ABG > 140 m/dl	0.92 (0.22-3.83)	0.910	0.71 (0.10-5.13)	0.732
	A/C > 1.22	1.04 (0.44-2.48)	0.926	0.95 (0.31-2.86)	0.923
	Thin-cap fibroatheroma				
	ABG > 140 m/dl	1.27 (0.31-5.29)	0.740	0.92 (0.12-6.82)	0.934
	A/C > 1.22	1.29 (0.55-3.00)	0.555	1.12 (0.36-3.44)	0.847

^∗^ *p* < 0.05; ^†^*p* < 0.01. ABG: acute blood glucose; A/C: acute versus chronic; CI: confidence interval; DM: diabetes mellitus; OR: odds ratio.

## Data Availability

The data that support the findings of this study are available from the corresponding author upon reasonable request.

## Supplementary Materials

Supplementary Materials Table S1: baseline characteristics of the study population and excluded patients. Table S2: linear regression analysis between A/C and cTnI/CK-MB. Figure S1: representative cross-sectional optical coherence tomography images.
