# Supplementary material for: Association between Admission Hyperglycemia and Culprit Lesion Characteristics in Nondiabetic Patients with Acute Myocardial Infarction: An Intravascular Optical Coherence Tomography Study
Source: J Diabetes Res. 2020 Jun 20;2020:1763567. doi: 10.1155/2020/1763567 (PMC7327614; doi:10.1155/2020/1763567)
Supplement: Supplementary Materials — Table S1: baseline characteristics of the study population and excluded patients. Table S2: linear regression analysis between A/C and cTnI/CK-MB. Figure S1: representative cross-sectional optical coherence tomography images. [file 1763567.f1.pdf]

SUPPLEMENTAL MATERIAL

Table S1 Baseline characteristics of the study population and excluded patients

	Included patients	Excluded patients	P value
Age, years	59.3 ± 11.8	57.4 ± 11.5	0.114
BMI, kg/m^2	26.2 ± 3.5	26.1 ± 3.7	0.771
Men	139 (88.5)	227 (81.9)	0.070
Smoking	122 (77.7)	192 (69.3)	0.060
Time from symptom onset, h	6.0 (2.9,13.8)	6.0 (2.9, 13.7)	0.553
Hypertension	102 (65.0)	162 (58.5)	0.184
Dyslipidemia	148 (94.3)	251 (90.6)	0.179
Diabetes mellitus	47 (29.9)	82 (29.6)	0.942
Prior PCI	38 (24.2)	21 (7.6)	<0.001
ABG, mg/dL	146.5 (126.2, 193.0)	142.6 (125.3, 190.8)	0.607
HbA1c, %	6.1 (5.7, 7.0)	5.9 (5.6, 7.0)	0.234
A/C	1.13 (1.01, 1.31)	1.15 (1.03, 1.32)	0.410
cTnI	3.7 ± 8.1	3.7 ± 8.4	0.950
CK-MB	65.8 ± 98.3	68.2 ± 103.6	0.807
WBC count, 10^6/L	10.8 ± 3.3	10.6 ± 3.1	0.652
Hemoglobin, g/L	150.2 ± 15.5	147.7 ± 16.4	0.132
TC, mg/dL	160.5 ± 38.2	171.0 ± 40.2	0.008
TG, mg/dL	140.9 ± 103.8	153.0 ± 108.5	0.256
HDL-C, mg/dL	42.9 ± 10.4	42.3 ± 12.9	0.655
LDL-C, mg/dL	98.4 ± 33.5	108.5 ± 33.2	0.003
Lp(a), mg/L	148.6 (69.8, 258.3)	164.0 (73.0, 367.0)	0.196
eGFR, mL/min/1.73 m2	93.3 ± 31.9	96.3 ± 31.4	0.347
Hs-CRP, mg/L	4.78 (2.38, 9.49)	5.78 (2.61, 10.74)	0.495
LVEF, %	54.0 ± 6.3	55.0 ± 6.2	0.088

Data shown are number (%), median (25th, 75th percentiles), or mean ± SD. ABG, acute blood glucose; A/C, acute/chronic; BMI, body mass index; CK-MB, creatine kinase MB; cTnI, cardiac troponin I; eGFR, estimated glomerular filtration rate; HbA1c, glycosylated hemoglobin; HDL-C, high-density lipoprotein cholesterol; Hs-

CRP, high-sensitivity C-reactive protein; LDL-C, low-density lipoprotein cholesterol; LVEF, left ventricular ejection fraction; PCI, percutaneous coronary intervention; TC, total cholesterol; TG, triglyceride; WBC, white blood cell.

Table S2 Linear regression analysis between A/C and cTnI/CK-MB

	β coefficients	95% confidence interval	p
For cTnI*			
A/C tertile	-0.76	-2.26 to 0.740	0.319
A/C level	-0.61	-4.30 to 3.07	0.744
For CK-MB*			
A/C tertile	-8.647	-36.72 to 9.43	0.346
A/C level	-7.16	-51.38 to 37.06	0.750

*Adjustment for time from symptom onset. A/C tertile was used as ordered categorical variable; A/C level was used as continuous carriable. A/C, acute-to-chronic glycemia ratio.

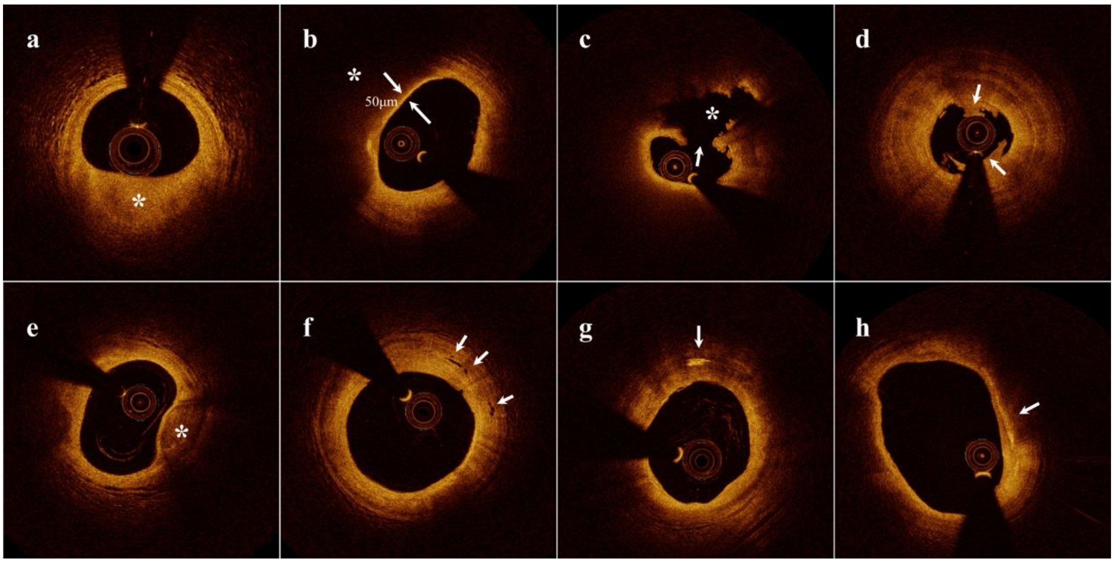

Figure S1. Representative cross-sectional optical coherence tomography images. **a** Fibrous plaque identified as a homogeneous, highly backscattering region (asterisk). **b** Lipid-rich plaque identified as a low-signal region with a diffuse border (asterisk) and thin-cap fibroatheroma with fibrous-cap thickness of 50μm. **c** Plaque rupture identified by disruption of the fibrous cap (arrow) and cavity formation (asterisk). **d** Plaque erosion identified by the presence of attached thrombus (arrow) overlying an intact plaque. **e** Calcification identified by the presence of a well-delineated, low-backscattering heterogeneous region (asterisk). **f** Microvessels defined as tubule luminal structures that do not generate a signal, with no connection to the vessel lumen (arrow). **g** Cholesterol crystal (arrow) identified by linear, highly backscattering structures without remarkable backward shadowing. **h** Macrophage infiltration (arrow) defined as a signal-rich, distinct, or confluent punctate region of higher intensity than background speckle noise that generates remarkable backward shadowing. Reprinted with permission from Sheng et al. (ref 18)
